# Supplementary material for: Evaluation of the Toxicity Potential of the Metabolites of Di-Isononyl Phthalate and of Their Interactions with Members of Family 1 of Sulfotransferases—A Computational Study
Source: Molecules. 2023 Sep 21;28(18):6748. doi: 10.3390/molecules28186748 (PMC10536557; doi:10.3390/molecules28186748)
Supplement: Supplementary file 1 [file molecules-28-06748-s001.zip › molecules-2612402-supplementary.pdf]

Evaluation of the toxicity potential of the metabolites of di-isononyl phthalate and their interactions with family 1 of sulfotransferases. A computational study.

Ceauranu Silvana, Ciorsac Alecu, Vasile Ostafe, Adriana Isvoran

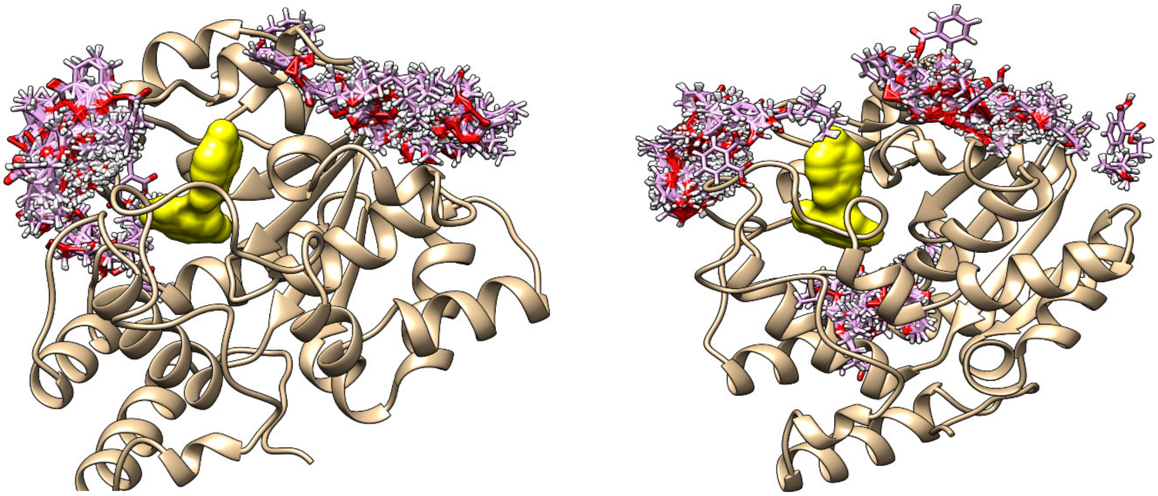

a b

Figure S1. Binding of mono-hydroxy-iso-nonyl phthalate **(a)** and mono-oxo-isononyl phthalate **(b)** to SULT1A1*1. SULT1A1*1 – brown ribbon, superposed molecules of p-nitrophenol – yellow surfaces, DiNP metabolites – stiks colored by atom type (C – purple, O – red, H – white).

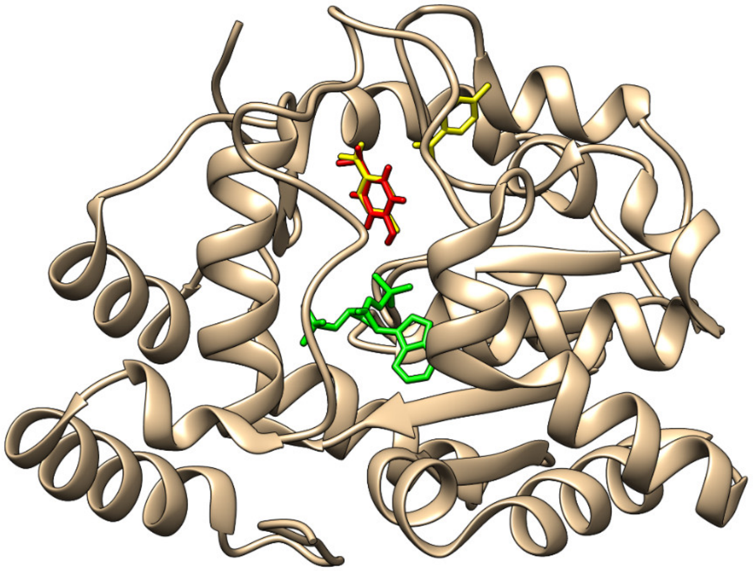

Figure S2. Outcome of the molecular docking study for docking p-nitrophenol to SULT1A1*2 compared with the crystalographic structure of the complex between SULT1A1*2a and p-nitrophenol. The position of p-nitrophenol in the cystalographic structure is revealed in yewllow sticks and the binding position resulting from docking study is revealed in red sticks. SULT1A1*2 enzyme is revealed as brown cartoon, the inactive cofactor adenosine-3'-5'-diphosphate is revealed in green sticks.

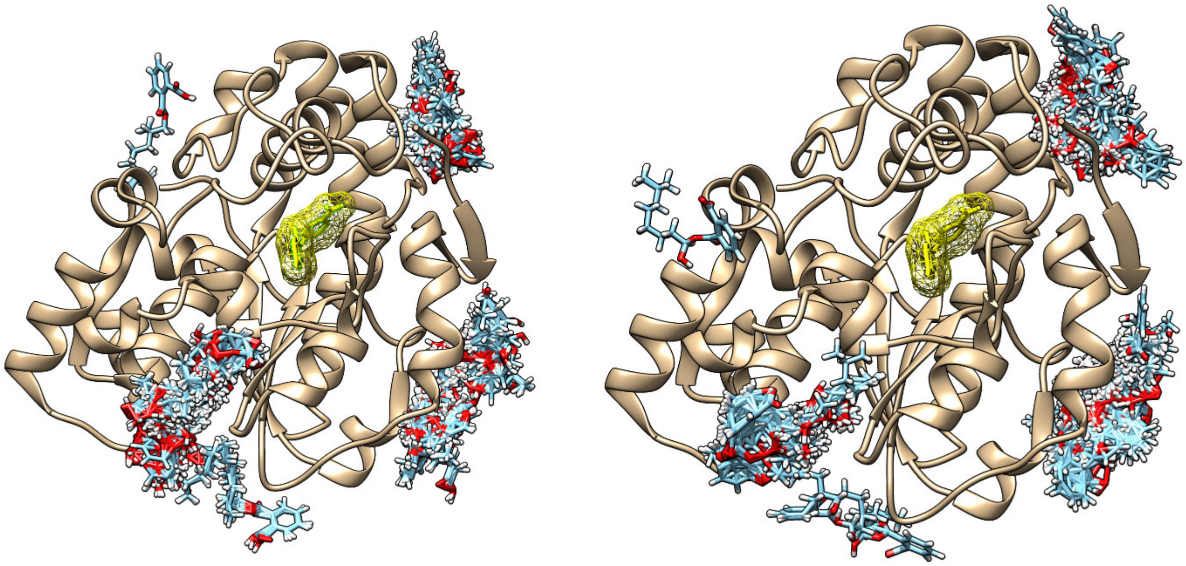

a **b**

Figure S3. Binding of mono-carboxy-iso-octyl phthalate **(a)** and mono-hydroxy-isononyl phthalate **(b)** to SULT1A1*2. SULT1A1*2 – brown ribbon, molecules of p-nitrophenol – yellow surfaces, DiNP metabolites – stiks colored by atom type (C – blue, O – red, H – white).

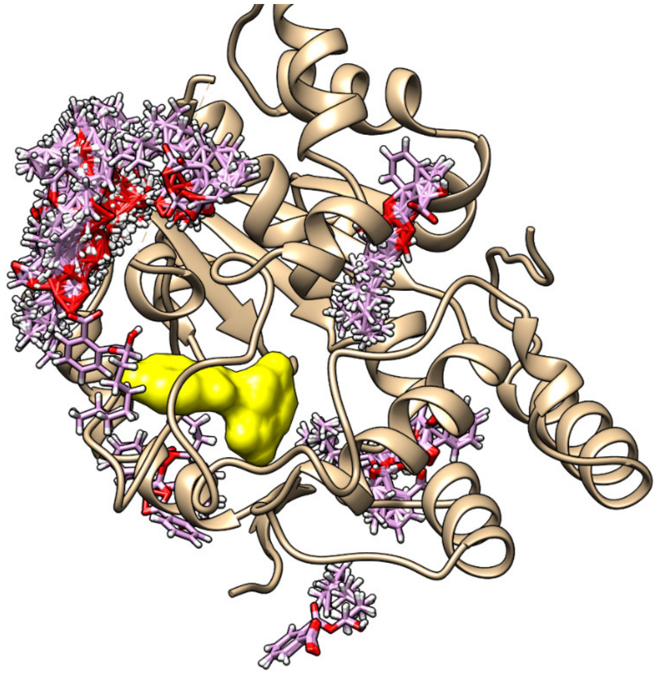

Figure S4. Binding of mono-hydroxy-iso-nonyl phthalate to SULT1A2. SULT1A2 – brown ribbon, p-nitrophenol – yellow surface, mono-hydroxy-iso-nonyl phthalate - stiks colored by atom type (C – purple, O – red, H – white).

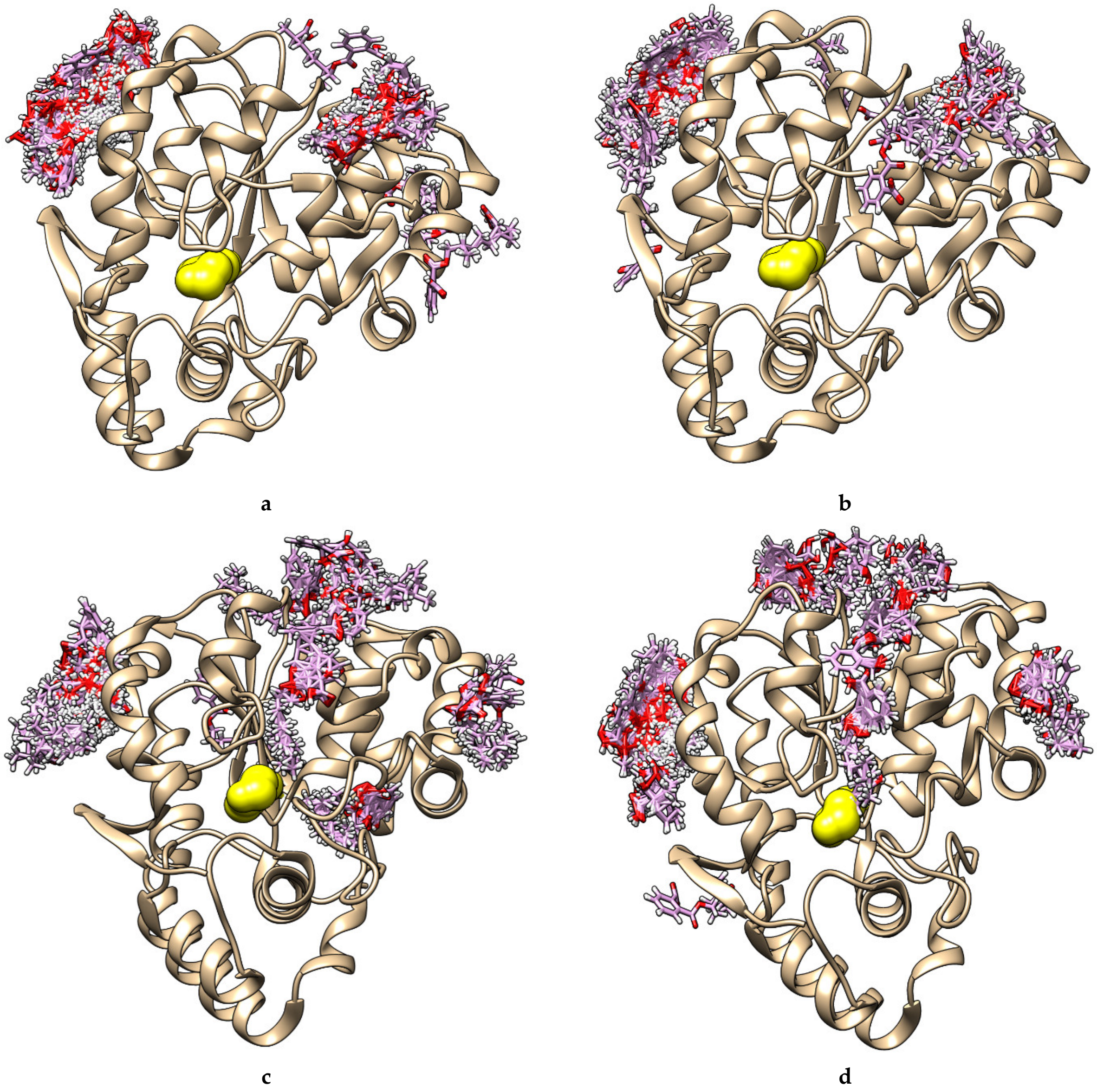

Figure S5. Binding of mono-carboxy-iso-octyl phthalate **(a)**, mono-hydroxy-iso-nonyl phthalate **(b)**, mono-isononyl phthalate **(c)**, and mono-oxo-isononyl phthalate **(d)** to SULT1A3. SULT1A3 – brown ribbon, dopamine – yellow surface, DiNP metabolites – stiks colored by atom type (C – purple, O – red, H – white).

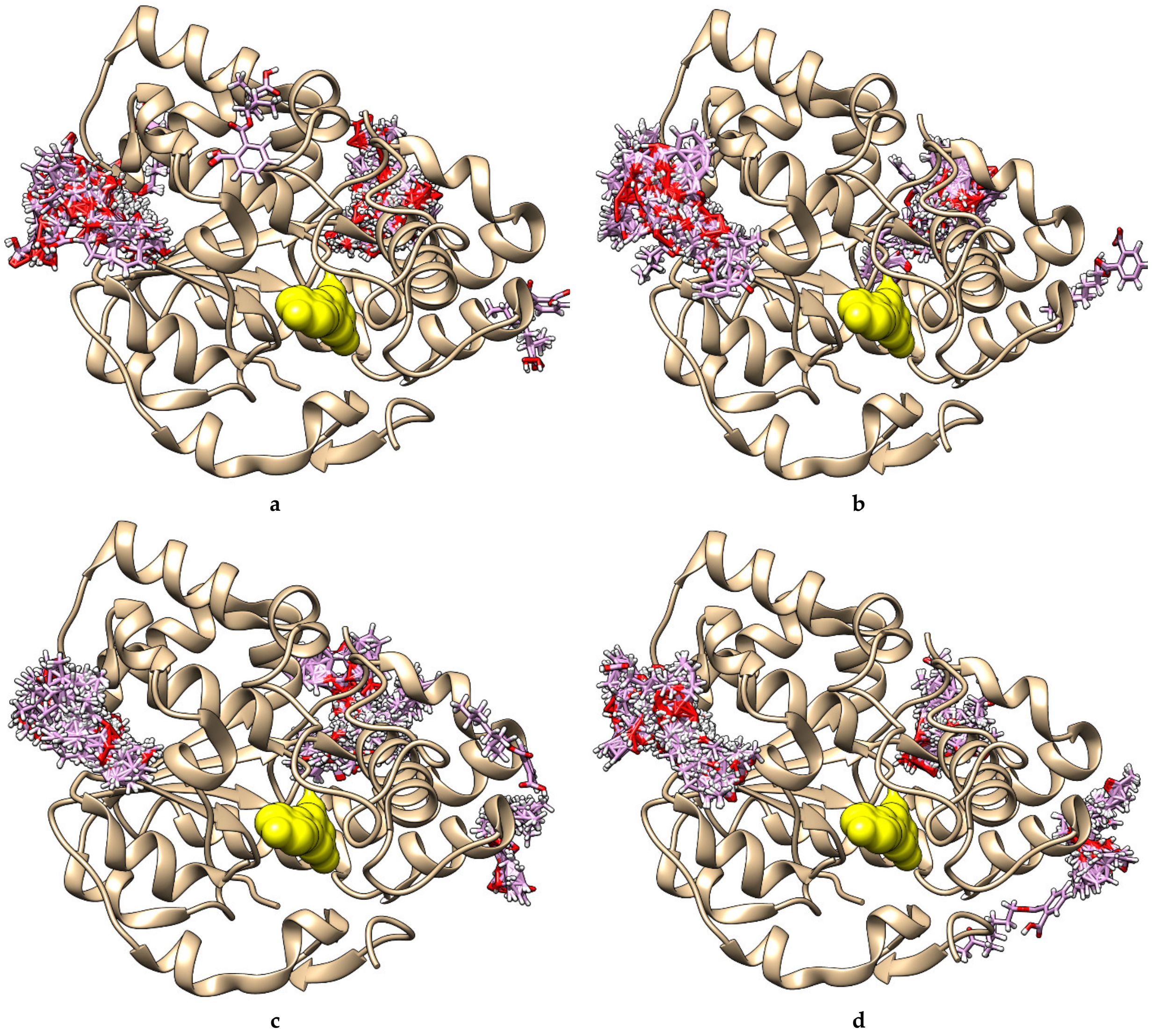

Figure S6. Binding of mono-carboxy-iso-octyl phthalate **(a)**, mono-hydroxy-iso-nonyl phthalate **(b)**, mono-isononyl phthalate **(c)**, and mono-oxo-isononyl phthalate **(d)** to SULT1B1. SULT1B1 – brown ribbon, resveratrol – yellow surface, DiNP metabolites – stiks colored by atom type (C – purple, O – red, H – white).

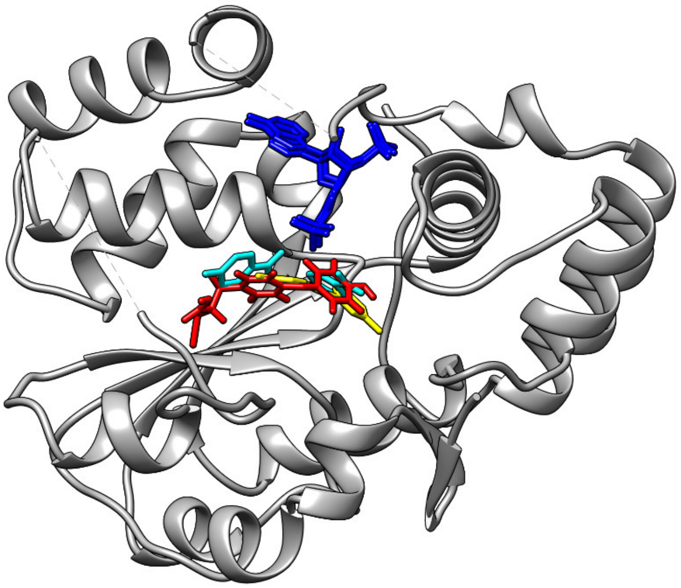

Figure S7. Superimposition of the outcome of the binding mode resulting from molecular docking of iodothyronine to SULT1C1 to the crystalographic structure of the complex between SULT1B1 and resveratrol and to the structure of SULT1A1*2 in complex with two molecules of p-nitrophenol. The positions of p-nitrophenol molecules are revealed in yellow sticks, that of resveratrol is revealed in cyan sticks and the binding position of iodothyronine resulting from docking study is revealed in red sticks. SULT1C1 enzyme is revealed as grey cartoon, the inactive cofactor adenosine-3'-5'-diphosphate is revealed in blue sticks.

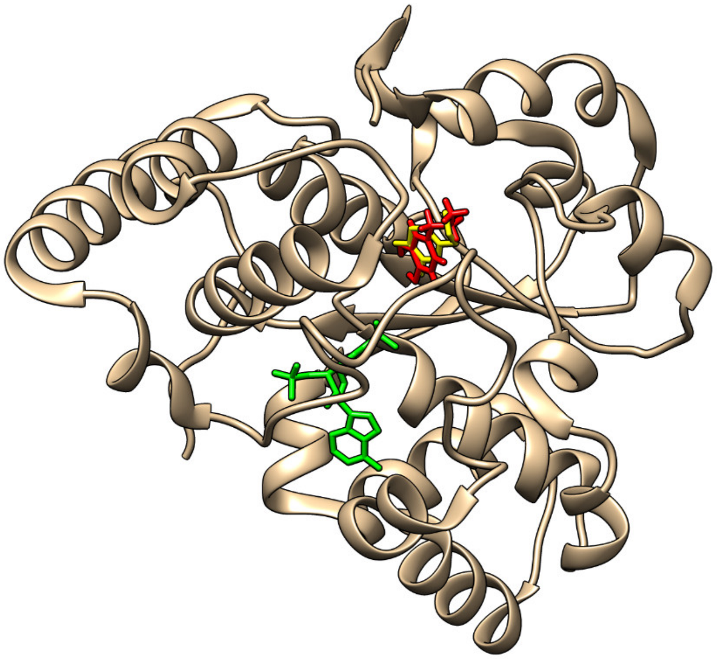

Figure S8. Outcome of the molecular docking study for docking dopamine to SULT1A3 compared with the crystalographic structure of the complex between SULT1A3 and dopamine. The position of dopamine in the cystalographic structure is revealed in yewllow sticks and the binding position resulting from docking study is revealed in red sticks. SULT1A3 enzyme is revealed as brown cartoon, the inactive cofactor adenosine-3'-5'-diphosphate is revealed in green sticks.

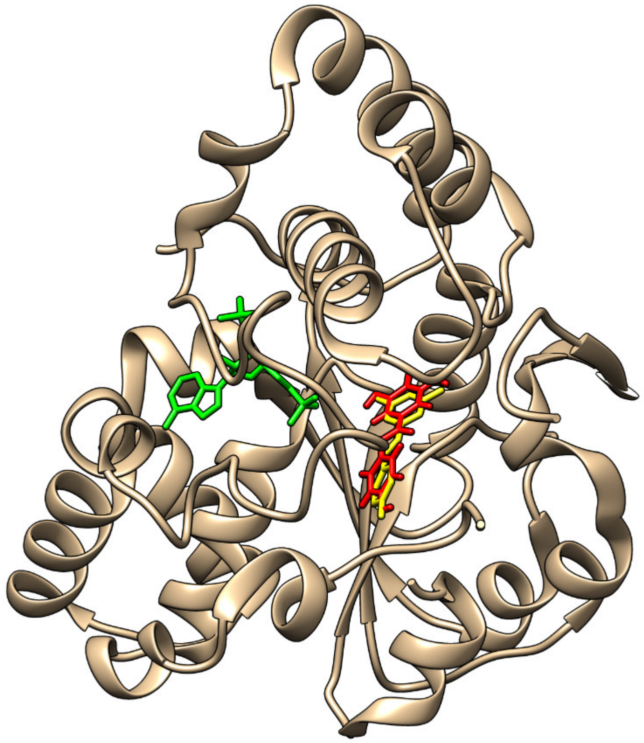

Figure S9. Outcome of the molecular docking study for docking resveratrol to SULT1B1 compared with the crystalographic structure of the complex between SULT1B1 and resveratrol. The position of resveratrol in the cystalographic structure is revealed in yewllow sticks and the binding position resulting from docking study is revealed in red sticks. SULT1B1 enzyme is revealed as brown cartoon, the inactive cofactor adenosine-3'-5'-diphosphate is revealed in green sticks.

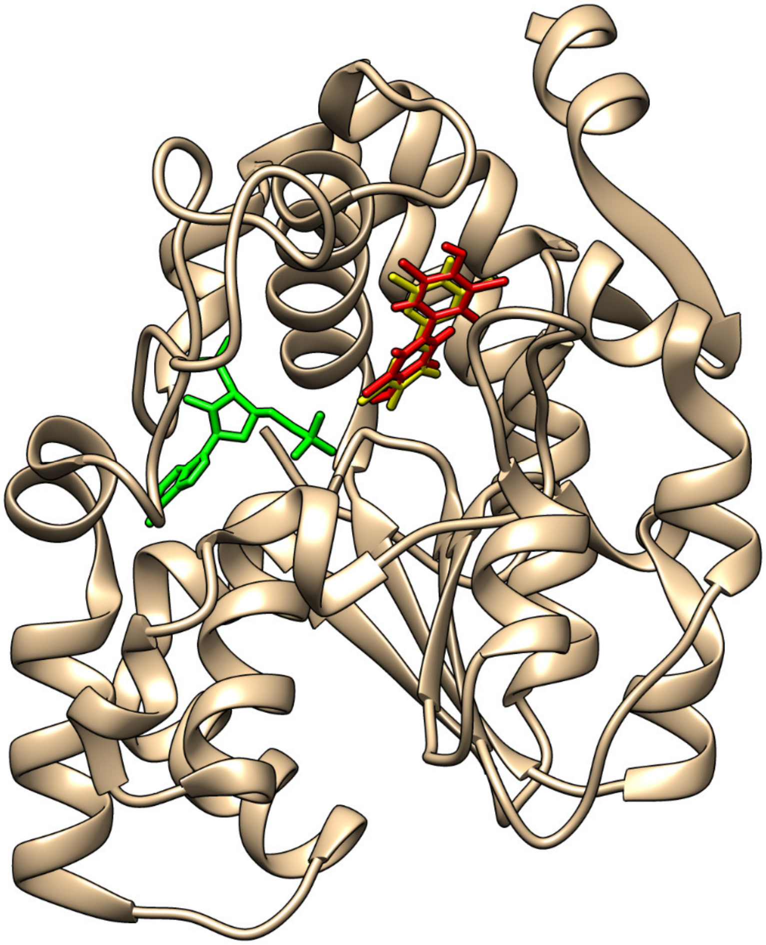

Figure S10. Outcome of the molecular docking study for docking the inhibitor 3,5,3',5'-tetrachloro-biphenyl-4,4'-diol to SULT1E1 compared with the crystalographic structure of the complex between SULT1E1 and 3,5,3',5'-tetrachloro-biphenyl-4,4'-diol. The position of the inhibitor 3,5,3',5'-tetrachloro-biphenyl-4,4'-diol in the cystalographic structure is revealed in yewllow sticks and the binding position resulting from docking study is revealed in red sticks. SULT1E1 enzyme is revealed as brown cartoon, the inactive cofactor adenosine-3'-5'-diphosphate is revealed in green sticks.

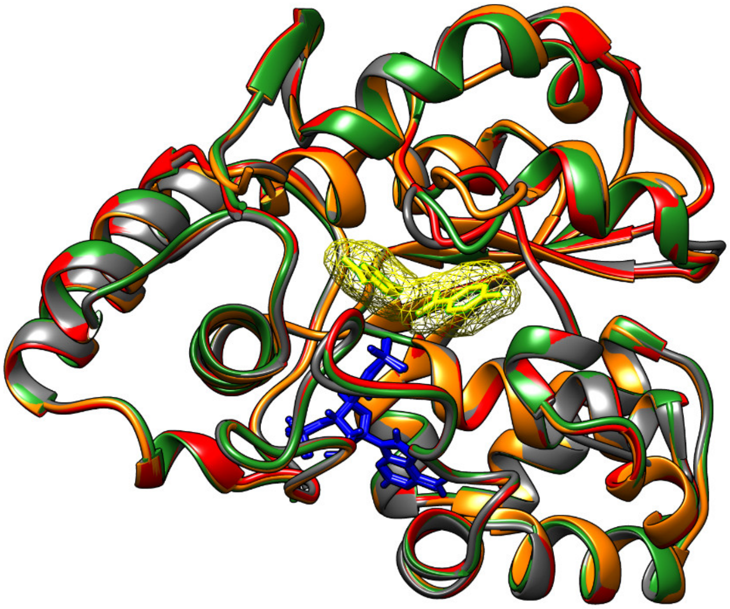

Figure S11. Superimposition of the structure of SULT1A1*2 (PDB ID 1LS6, red ribbon) with structures of SULT1A1*1 (PDB ID 4GRA, forest green ribbon, RMSD=0.298 Å for 288 pruned carbon alpha atoms pairs), SULT1A1*3 (PDB ID 1Z28, RMSD=0.367 Å for 288 pruned carbon alpha atoms pairs) and SULT1A2 (PDB ID 1Z29, RMSD=0.319 Å for 288 pruned carbon alpha atoms pairs). The position of the inactive cofactor adenosine-3'-5'-diphosphate is similar in all the structural files and is reveled in blue sticks. The position of the two molecules of p-nitrophenol that are present in the structural file of SULT1A1*2 is revealed in yellow sticks and mesh surface.

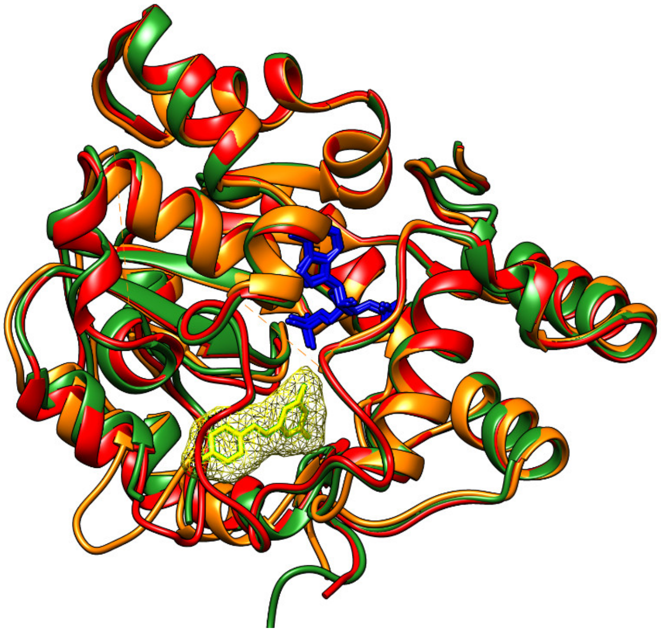

Figure S12. Superimposition of the structure of SULT1C1 (PDB ID 3BFX, orange ribbon) with structure of SULT1B1 (PDB ID 3CKL, forest green ribbon, RMSD=0.837 Å for 219 pruned carbon alpha atoms pairs) and with structure of SULT1A1*2 (PDB ID 1LS6, red ribbon, RMSD=0.857 Å for 220 pruned carbon alpha atoms pairs). The position of the inactive cofactor adenosine-3'-5'-diphosphate is similar in both structural files and is reveled in blue sticks. The position of resveratrol that is present in the structural file of SULT1B1 is revealed in yellow sticks and mesh surface.

Table S1. Physicochemical and structural properties of the metabolites of di-isononyl phthalate and of the ligands that are present in the crystallographic structures of SULT1 enzymes: MW – molecular weight, logP –partition coefficient, HBD – hydrogen bonds donors, HBA – hydrogen bonds acceptors, RB – rotatable bonds, tPSA – topological polar surface area.

Compound	MW (g/mol)	LogP	HBD	HBA	RB	tPSA (Å^2)	Area (Å^2)	Volume (Å^3)
resveratrol	228.24	3.1	3	3	2	60.7	217.7	191.0
monohexyl phthalate	250.29	4.2	1	4	8	63.6	246.9	217.8
monooctyl phthalate	278.34	5.3	1	4	10	63.6	283.2	245.9
Monoethylhexyl phthalate	278.34	4.4	1	4	9	63.6	277.2	252.4
monobutyl phthalate	222.24	3.1	1	4	6	63.6	214.3	189.0
mono-cyclohexyl phthalate	248.47	2.9	1	4	4	63.6	226.0	210.9
